# Relationship between Expression of the Family of M Proteins and Lipoteichoic Acid to Hydrophobicity and Biofilm Formation in *Streptococcus pyogenes*

**DOI:** 10.1371/journal.pone.0004166

**Published:** 2009-01-09

**Authors:** Harry S. Courtney, Itzhak Ofek, Thomas Penfound, Victor Nizet, Morgan A. Pence, Bernd Kreikemeyer, Andreas Podbielbski, David L. Hasty, James B. Dale

**Affiliations:** 1 Department of Medicine, University of Tennessee, Memphis, Tennessee, United States of America; 2 Department of Anatomy and Neurobiology, University of Tennessee, Memphis, Tennessee, United States of America; 3 Department of Molecular Sciences, University of Tennessee, Memphis, Tennessee, United States of America; 4 Department of Veterans Affairs Medical Center, University of Tennessee, Memphis, Tennessee, United States of America; 5 Department of Clinical Microbiology and Immunology, Sackler Faculty of Medicine, Tel Aviv University, Tel Aviv, Israel; 6 Department of Pediatrics, University of California San Diego, La Jolla, California, United States of America; 7 Department of Medical Microbiology and Hospital Hygiene, Hospital of Rostock University, Rostock, Germany; Alfa Institute of Biomedical Sciences (AIBS), Greece

## Abstract

**Background:**

Hydrophobicity is an important attribute of bacteria that contributes to adhesion and biofilm formation. Hydrophobicity of *Streptococcus pyogenes* is primarily due to lipoteichoic acid (LTA) on the streptococcal surface but the mechanism(s) whereby LTA is retained on the surface is poorly understood. In this study, we sought to determine whether members of the M protein family consisting of Emm (M protein), Mrp (M-related protein), Enn (an M-like protein), and the streptococcal protective antigen (Spa) are involved in anchoring LTA in a manner that contributes to hydrophobicity of the streptococci and its ability to form biofilms.

**Methodology/Principal Findings:**

Isogenic mutants defective in expression of *emm*, *mrp*, *enn*, and/or *spa* genes of eight different serotypes and their parental strains were tested for differences in LTA bound to surface proteins, LTA released into the culture media, and membrane-bound LTA. The effect of these mutations on the ability of streptococci to form a hydrophobic surface and to generate biofilms was also investigated. A recombinant strain overexpressing Emm1 was also engineered and similarly tested. The serotypes tested ranged from those that express only a single M protein gene to those that express two or three members of the M protein family. Overexpression of Emm1 led to enhanced hydrophobicity and biofilm formation. Inactivation of *emm* in those serotypes expressing only a single *emm* gene reduced biofilm formation, and protein-bound LTA on the surface, but did not alter the levels of membrane-bound LTA. The results were more varied in those serotypes that express two to three members of the M protein family.

**Conclusions/Significance:**

Our findings suggest that the formation of complexes with members of the M protein family is a common mechanism for anchoring LTA on the surface in a manner that contributes to hydrophobicity and to biofilm formation in *S. pyogenes*, but these activities in some serotypes are dependent on a trypsin-sensitive protein(s) that remains to be identified. The need for interactions between LTA and M proteins may impose functional constraints that limit variations in the sequence of the M proteins, major virulence factors of *S. pyogenes*.

## Introduction

The hydrophobic properties of bacterial surfaces are a major determinant in the adhesion of bacteria and in the formation of biofilms by bacteria on animate and inanimate surfaces [1]. Hydrophobicity is likely due to a complex interplay between negatively-charged, positively-charged, hydrophobic and hydrophilic components on the surface of the bacteria. Surface components that directly contribute to hydrophobicity have been termed hydrophobins and this contribution can be measured in a number of ways including the MATH (microbial adhesion to hydrocarbons) test [1]. Lipoteichoic acid (LTA) is a major hydrophobin that contributes to the hydrophobicity of a variety of Gram-positive bacteria [1], [2], [3], [4].

LTA also plays important roles in bacterial physiology by chelating metals, maintaining the integrity of the membrane and controlling autolytic enzymes [5], [6]. Purified LTA has been shown to exhibit a number of biological effects including bone resorption and activation of cells of the immune system such as macrophages and T cells [5], [7], [8], [9]. Expression of LTA is vital to Gram-positive bacteria; bacteria that are completely devoid of LTA cannot grow [10] and bacteria with reduced expression of LTA are deficient in one or more of the functions mentioned above [3].

In *Streptococcus pyogenes*, LTA functions not only as a hydrophobin but it also mediates adhesion of the organisms to a variety of host cells [2], [11]. The hydrophobicity of a number of serotypes of *S. pyogenes* is dependent upon the expression of surface proteins that form complexes with LTA in a manner that allows the ester linked fatty acids of LTA to be exposed on the bacterial surface [12]. These fatty acids mediate the binding of LTA to receptors of host cells and the inhibition of streptococcal adhesion to host cells [11], [13]. Thus, LTA complexed on the streptococcal surface via interactions with proteins are involved in adhesion to host cells.

**Figure 1:**
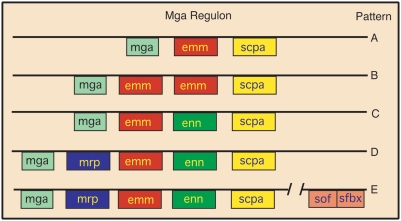
Schematic of Mga regulon patterns. Mga (multigene activator) is a positive regulator of a number of streptococcal genes. The most prominent of these are the family of M proteins whose genes are tandemly linked. *sof* and *sfbx* are bicistronic and are also regulated by Mga, but are located some distance away. *emm* encodes for M protein, *mrp* encodes M-related proteins, *enn* encodes an M-like protein that binds IgA, and *scpa* encodes a C5a peptidase. Some serotypes contain only *mga*, *emm*, and *scpa* (pattern A). Other serotypes contain one or more of the remaining genes (patterns B–E). The M1, M5, M6 and M24 strains in this work are pattern A; the M18 strain is pattern C; and the M2, M4, and M49 strains are pattern E. Figure derived from data and classification scheme of Bessen at al. [14], [15], [16].

The M protein family comprises a major group of proteins on the surface of *S. pyogenes* that consists of M protein (Emm, note that the terms M protein and Emm are used interchangeably), M-related protein (Mrp), and Enn (another M-like protein). Some serotypes express only Emm (pattern A, Fig. 1), while other serotypes express Mrp and/or Enn in addition to Emm (patterns C, D, and E) [14], [15], [16]. M protein has been previously identified as a major surface protein that was able to form complexes with LTA [17]. However, these experiments were performed with only one serotype that expressed only a single member of the M protein family (i.e., pattern A) and the possibility that LTA might form complexes with other proteins was not investigated. Therefore, the present investigation was undertaken to determine if M protein from other serotypes and other members of the M protein family may also be involved in anchoring LTA to the surface, thereby contributing to hydrophobicity and to the formation of biofilms. For these studies we used isogenic mutants lacking one or more of members of the M protein family and another surface protein that is involved in resistance to phagocytosis, the streptococcal protective antigen or Spa [18], [19]. Our findings indicate that members of the M protein family are centrally involved in the formation of a hydrophobic cell surface and in biofilm formation in most serotypes. However, we also determined that one or more additional trypsin-sensitive proteins are involved in these functions in some serotypes and these proteins remain to be identified.

## Materials and Methods

### Bacterial strains and growth conditions

The sources of the parental strains employed and their isogenic mutants are listed in Table 1. That inactivation of the targeted genes resulted in loss of expression of those genes has been described (see references in Table 1). The strains used in this investigation are clinical isolates. The M1 strain used in this investigation was the M1T1 clone that has disseminated globally and is the single leading cause of group A streptococcal invasive infections such as necrotizing fasciitis or toxic shock syndrome, and is also the most common cause of pharyngitis. The strains were grown overnight at 37°C in Todd-Hewitt broth supplemented with 0.5% yeast extract (THY) unless noted otherwise. THY was supplemented with the protease inhibitor E64 (1 µg/ml) (Sigma, St. Louis, MO) in some experiments to determine hydrophobicity. The bacteria were harvested, washed twice in distilled water and suspended in either water or in PBS (0.01 M phosphate, 0.14 M NaCl, pH 7.4) to the desired optical density and kept on ice until further testing. The culture supernatant was passed through a 0.2 µm pore size filter, heated for 10 minutes at 95°C and kept in the cold until used.

**Table 1:** Biofilms, hydrophobicity, and surface, membrane-bound, and culture released LTA of *Streptococcus pyogenes*

M type and gene targeted for mutation	Biofilm formation, percent of control^a^	Hydro-phobicity, percent of control^b^	Protein-bound LTA released by trypsin, µg^c^	Membrane-bound LTA phenol extracted, µg^d^	LTA released in culture medium, µg/ml	Reference for strains
M1 wt	100±12	100±1	4.8±1.1	29.0±7.8	7.5±1.5	[44]
ΔEmm1	56±14*	22±6*	1.7±1.0*	29.6±7.6	6.8±1.3	submitted^e^
M5 wt	100±6	100±1	5.8±1.3	27.0±7.6	7.3±1.5	This study
ΔEmm5	4±4*	34±6*	1.9±1.3*	23.8±10.3	5.1±0.4*	This study
M6 wt	100±3	100±5	9.5±2.5	47.6±1.7	7.0±1.4	[45]
ΔEmm6	46±8*	8±2*	2.6±0.7*	47.7±5.2	8.0±2.2	[45]
M24 wt	100±35	100±1	7.8±1.7	28.9±4.9	5.0±0.7	[46]
ΔEmm24	5±3*	19±30*	5.0±1.0*	23.5±4.7	8.2±1.6*	[46]
M18 wt	100±11	100±1	7.7±1.2	52.1±11.9	6.5±1.1	[18]
ΔEmm18	118±15	72±11	8.8±1.8	51.5±2.8	6.0±2.3	[18]
ΔSpa18	58±3*	114±1	7.0±2.5	49.4±4.9	8.4±2.2	[18]
ΔEmm18/Spa	62±4*	109±22	9.8±2.8	49.4±0.5	8.8±1.9	[18]
M2 wt	100±5	100±3	7.3±0.1	54.0±2.9	7.3±1.6	[35]
ΔEmm2	79±8*	25±3*	8.0±1.5	54.5±2.5	5.6±0.5	[35]
ΔMrp2	94±5	95±5	7.3±0.2	56.8±5.2	8.2±1.4	[35]
M4 wt	100±15	100±1	11.3±0.1	57.0±3.3	9.5±3.4	[36]
ΔEmm4	118±6	56±1*	13.5±0.1	57.9±5.7	9.0±1.9	[36]
ΔMrp4	4±6*	22±3*	5.8±0.2*	52.8±12.7	7.9±1.6	[36]
ΔEnn4	116±2	75±5*	7.6±2.3*	56.5±4.1	9.4±2.8	[36]
M49 wt	100±8	100±2	7.1±1.0	56.0±5.6	9.5±1.5	[35]
ΔEmm49	70±15*	83±7	7.8±1.9	57.4±7.4	10.2±2.7	[35]
ΔMrp49	44±16*	98±4	7.2±0.4	54.4±6.9	9.1±3.4	[35]

a biofilm formation was determined in microtiter wells as described in Materials&Methods, biofilm formation by parental strains (wt) was the positive control and set at 100%.

b hydrophobicity was determined by adhesion of streptococci to hexadecane as described in Materials&Methods, adhesion by parental strains was the control and set at 100%.

c µg of LTA complexed to surface proteins was obtained by trypsin extraction of 1 ml of streptococci (OD_530_ = 1.0) as described in Materials&Methods.

d µg of LTA obtained by phenol extraction of 1 ml of trypsinized streptococci (OD_530_ = 1.0) as described in Materials&Methods.

e Lauth, X., M. von Kockritz-Blickwede, C.W. McNamara, S. Myskowski, A.S. Zinkernagel, B. Beall, P. Ghosh, R. L. Gallo, and V. Nizet. 2008. M1 protein allows group A streptococcal survival in phagocyte extracellular traps by cathelicidin inhibition. (submitted).

* indicates that the mutant is significantly different from the parent, p≤0.05 as determined with students t-test.

All experiments were performed in triplicate. The mean±SD is shown.

### Inactivation of the *emm* gene in M type 5 *S. pyogenes*

The *emm* gene was inactivated by allelic replacement in the M type 5 *S. pyogenes* strain Manfredo. Mutagenic primers were used to create a stop codon at base pair 60 of the sequence encoding the mature M5 protein. The modified gene was ligated into pGHost+9 vector and electroporated into the streptococci. Procedures for antibiotic and temperature selection for single- and double-crossover events were as described by Maguin et al. [20]. Confirmation that the wild type gene was replaced by the mutagenized *emm* gene was confirmed by PCR and sequencing. Western blot analysis of hot acid extracts of parent and mutant strains confirmed that Emm5 was not expressed in the mutant. Hot acid treatment is a classical method for the extraction of M proteins [21].

### Construction of recombinant strain overexpressing Emm1

Primers were designed to amply the DNA sequence encoding Emm1. The PCR product was ligated into the expression vector pDCerm [22], [23] to obtain the plasmid p*emm1* that was electroporated into the M type 1 strain 5448. An erythromycin resistant colony was selected for further studies and labeled M1+p*emm1*.

### Enzyme linked immunosorbent assays

The streptococci were grown overnight in THY at 37°C except for the recombinant strain M1+p*emm1*, which was grown in THY containing 5 µg/ml erythromycin. The streptococci were washed in PBS, adjusted to an OD_530_ of 0.4 and 100 µl was added to microtiter wells. The plates were incubated at 37°C for 30 min and non-adherent bacteria were removed by washing. The wells were then blocked with 1% bovine serum albumin (BSA) for 30 min at 37°C. A 1∶1000 dilution of normal rabbit serum (NRS) or rabbit serum against a synthetic peptide copying amino acid residues 1–26 of the mature Emm1 was added to the appropriate wells. The production of anti-SM1(1–26) has been described [24]. After a 30 min incubation at 37°C, the wells were washed and 100 µl of a 1∶2000 dilution of peroxidase-labeled goat anti-rabbit immunoglobulins was added to all wells and incubated for 30 min at 37°C. The wells were then washed and 100 µl of the tetramethylbenzidine was added. Stop solution was added after color development and the A_450_ was recorded. Wells coated with BSA served as blanks. The values obtained with NRS were considered non-specific and were subtracted from values obtained with anti-SM1(1–26). Assays were done in triplicate.

### Biofilm assays

A microtiter method described by several investigators to measure biofilm formation by *S. pyogenes* was used with minor modifications [25], [26], [27]. Briefly, the streptococci were grown overnight in THY (unless stated otherwise), diluted 1∶40 in THY, and 100 µl of the suspension added to wells of a polystyrene, microtiter plate (Corning Costar 3595, Fisher Scientific, Pittsburg, PA). In some instances the growth media was supplemented with 1 mg/ml of trypsin. The plates were incubated in a humidified environment for 48 hours at 37°C. Afterwards, the plates were gently washed four times with PBS, any residual fluid was carefully removed, and the plates were heat fixed at 60°C for 1 hour. Hucker's crystal violet solution (100 µl) was added to each well. After two minutes, the wells were washed with tap water until the water became clear. Then, 100 µl of destain solution (10% methanol, 7.5% acetic acid in distilled water) was added, the plates were shaken for 1 minute, and the absorbance at 540 nm was recorded. Wells incubated with THY without streptococci were used as blanks. Additional controls consisted of measuring the OD_530_ of the streptococci after an overnight growth in THY with or without trypsin to verify that trypsin treatment did not alter growth.

The bacteria were also grown at 37°C in 100% normal rabbit serum, trypticase soy broth without glucose (TSB), TSB+1% glucose, Mueller Hinton II medium (MHII), Luria-Bertani broth (LB), and THY and then treated as described above to determine the effect of growth media on biofilm formation. All growth media were purchased from Difco Labs except TSB without glucose, which was purchased from Sigma Chem. Co.

### Determination of hydrophobicity

Hydrophobicity was determined by the hexadecane method as described [12]. Briefly, 1 ml of bacteria (OD_530_ = 1.0) was placed into glass tubes and 100 µl of hexadecane (Sigma, St. Louis, MO) was added. The mixtures were vigorously vortexed for 2 minutes, followed by 10 min incubation at ambient temperature to allow phase separation, and then the OD_530_ of the lower, aqueous phase was recorded. In some cases, the bacteria were treated with bovine testicular hyaluronidase (Sigma, St. Louis, MO) at 2 µg/ml at 37°C for 15 min and then tested for adhesion to hexadecane. The percent hydrophobicity was calculated by the formula: % hydrophobicity = [1−(OD_530_ after vortexing/OD_530_ before vortexing)]×100.

### Enzymatic treatment and phenol extraction of the bacteria

Forty µl of trypsin (1 mg/ml PBS, Sigma, St. Louis, MO) was added to 0.4 ml bacterial suspension (OD_530_ = 1.0) in PBS. After 30 minutes at 37°C, the bacteria were centrifuged and the supernatant was removed, heated to 95°C for 5 min to inactivate enzymes, and saved for determinations of LTA content. The sedimented bacteria were washed twice with distilled water and then assayed for hydrophobicity.

After trypsinization, LTA was extracted from the streptococci with phenol as previously described [28] by adding an equal volume of phenol (Sigma, St. Louis, MO) heated to 60°C to a streptococcal suspension that had been adjusted to an OD_530_ of 1.0 in distilled water and then vortexing for 5 min. The mixtures were then centrifuged at 14,000×g. The aqueous phase was removed and dialyzed extensively against distilled water. This extract represents membrane-bound LTA.

### Quantitation of LTA

LTA concentrations were determined by a competitive ELISA as described previously with slight modification [29]. Microtiter wells were coated with commercial LTA (Sigma, St. Louis, MO) by adding 100 µl of LTA (5 µg/ml in carbonate buffer), and incubating the plate for 1 hour at 37°C. The wells were washed with PBS containing 0.05% Tween-20 (PBST), then blocked with 5% v/v hemoglobin in PBST for 30 minutes at 37°C. LTA standards were made with either commercial LTA or highly purified LTA extracted from streptococci with butanol, as described previously [8]. 50 µl of serial two-fold dilutions of LTA standards or two-fold dilutions of culture supernatants, trypsin-released LTA, or phenol-extracted LTA were added to 50 µl of a 1∶3200 dilution of a mouse monoclonal antibody against LTA (Abcam, Cambridge, MA). After incubating for 30 min at ambient temperature, the mixtures were added to the LTA-coated wells and incubated for 60 minutes at 37°C. After washing, 100 µl of HRP-conjugated, goat anti-mouse immunoglobulin (MP Biomedicals, Solon, OH) diluted 1∶2500 was added and incubated for 1 hour at 37°C. The plates were then washed and the turbo TMB substrate (Pierce Chem Co., Rockford, IL) was added. A stop solution (sulfuric acid) was added after color development and the A_450_ recorded. The ELISA values of negative control wells not coated with LTA were subtracted from all experimental values. The amount of LTA in samples was determined from standard curves. All measurements were performed in triplicate.

The amount of LTA needed to achieve 50% inhibition was 3.1 µg/ml for butanol extracted LTA and 5.1 µg/ml of commercial LTA. This indicated that about 40% by weight of the commercial LTA obtained from Sigma was not immunoreactive with the monoclonal antibody against polyglycerol phosphate. These results are in agreement with those of Morath et al. who also determined the purity of commercially acquired LTA [30]. Therefore, a correction factor of 0.60 was applied to results from assays that used commercial LTA as standards.

## Results

### Biofilm formation

**Figure 2:**
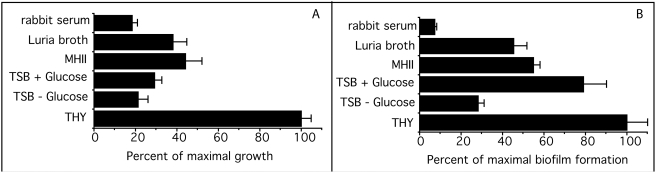
Effect of growth medium on biofilm formation. A. The growth of M type 4 *S. pyogenes* in the indicated media was determined by measuring the A_530_ after a 48 hour incubation at 37°C. B. The formation of biofilms by M type 4 *S. pyogenes* grown for 48 hours at 37°C in the indicated media. These experiments were done in quadruplicate and the mean±SD is shown.

To determine the optimal medium for biofilm formation, we tested the ability of streptococci to grow and form biofilms in various media (Fig. 2). The addition of glucose to TSB had a minor effect on growth but enhanced biofilm formation by approximately threefold. THY provided the best medium for growth and for biofilm formation and, therefore, was used for all subsequent experiments.

**Figure 3:**
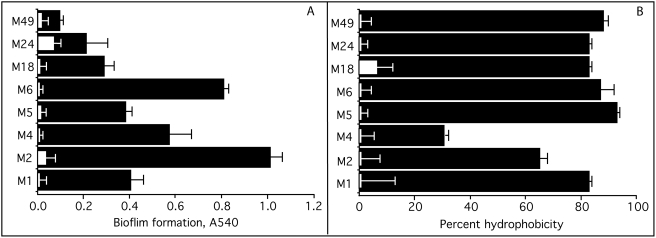
Biofilm formation and hydrophobicity of *S. pyogenes*. A. Biofilm formation by the various serotypes was determined by the microtiter assay as described in Materials&Methods. Black bars indicate biofilm formation by various serotypes and white bars indicate formation of biofilms in the presence of trypsin. B. Hydrophobicity of *S. pyogenes*. Black bars indicate the degree of hydrophobicity of the various serotypes as measured by their ability to bind to hexadecane. White bars indicate the percent hydrophobicity after trypsin treatment. All experiments were done in triplicate and performed at least twice. The mean±SD is shown.

There was significant variation among different M types in their ability to form biofilms (Fig. 3A). These findings were similar to those of Lembke et al al. [26] and Baldassarri et al. [27] who also found that biofilm formation varied among different serotypes and even among strains of the same M type. We found that M types 2 and 6 exhibited the greatest degree of biofilm formation and M type 49 exhibited the least degree. Similar findings have been reported regarding M types 6 and 49 *S. pyogenes* [26], [27]. The addition of trypsin to the growth media blocked the formation of biofilms by all of serotypes tested indicating the dependence of biofilms on streptococcal surface proteins (Fig. 3A). In control assays trypsin did not alter the growth of the streptococci. This finding is in agreement with those of others [31], [32] who also found that trypsin did not alter the viability and growth of group A streptococci.

**Figure 4:**
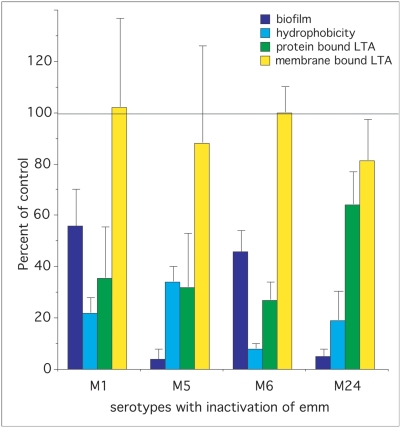
Effect of inactivation of *emm* in pattern A serotypes on biofilm formation, hydrophobicity, and LTA expression. The values for the wild type parental strain was used as the control and set at 100% in each case and data for the mutants are provided as percent of control (data from table 1). The horizontal line at the 100% mark represents the values for parental controls, which are not individually shown.

**Figure 5:**
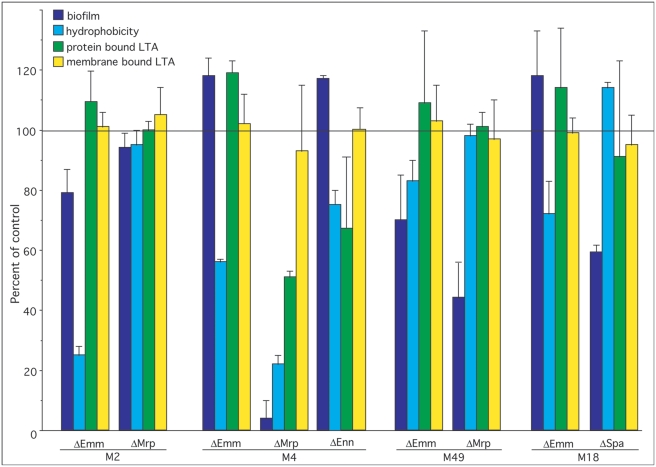
Effect of inactivation of *emm*, *mrp*, *enn* or *spa* in patterns C, D, and E serotypes on biofilm formation, hydrophobicity, and LTA expression. The values for wild type parental strain was used as the control and set at 100% and the data for mutants are provided as percent of control (data from table 1). The horizontal line at the 100% mark represents the values for parental controls, which are not individually shown.

To investigate the role that various members of the M protein family play in the formation of biofilms, we compared the ability of parental strains to make biofilms to that of mutants defective in the expression of Emm, Mrp, Enn, and Spa (Table 1, Fig. 4&5). The data indicated that Emm-negative mutants from all of the pattern A serotypes (i.e. M1, M5, M6, and M24) were deficient in their ability to form biofilms (Fig. 4). In M type 2, ablation of *emm2* had only a minor effect and ablation of *mrp2* had no significant effect (Fig. 5). However, in M type 4 ablation of *mrp4* reduced biofilms by 96%, whereas disruption of *emm4* and *enn4* was without effect (Fig. 5). In M type 49, inactivation of Emm49 and Mrp49 reduced biofilms by 30% and 56% respectively (Fig. 5). In the one pattern C serotype tested (M18), Spa seemed to play a significant role as inactivation of *spa* resulted in a 42% decrease in its biofilm (Fig. 5).

### Hydrophobicity

The adhesion of streptococci to hexadecane was used to measure hydrophobicity of eight different serotypes of *S. pyogenes* (Fig. 3B). The degree of hydrophobicity was not as variable as the ability to form biofilms. However, M type 4 *S. pyogenes* was significantly less hydrophobic than the other serotypes tested. Previous work indicated that expression of the hyaluronate capsule interferes with the hydrophobicity of *S. pyogenes* [12]. Therefore, M type 4 *S. pyogenes* was treated with hyaluronidase and then tested for adhesion to hexadecane. Adhesion to hexadecane increased from 31% in the untreated control to 64% after hyaluronidase treatment, indicating that the capsule was largely responsible for the low adhesion of the M4 serotype to hexadecane.

To determine if surface proteins participated in the adhesion of these serotypes to hexadecane, the streptococci were treated with trypsin and then tested for binding to hexadecane (Fig. 3B). Trypsin treatment almost completely abolished the ability of these streptococci to adhere to hexadecane suggesting that proteins contribute significantly to the hydrophobicity of these streptococci. Next, we utilized defined mutants to determine whether members of the M protein family are involved in adhesion to hexadecane (Table 1, Fig. 4&5). As noted with biofilm formation, inactivation of *emm* genes in pattern A serotypes (M1, M5, M6, and M24) dramatically reduced their hydrophobicity (Fig. 4). Also inactivation of *emm* in M type 2 reduced hydrophobicity (Fig. 5). In M type 4, all three members of the M protein family contributed to hydrophobicity in the order of Mrp4>Emm4>Enn4 (Fig. 5). Inactivation of Emm18 reduced hydrophobicity by 38%. In contrast, none of the mutations tested had any significant effect on the hydrophobicity of M type *49 S. pyogenes* (Fig. 5).

### Expression of LTA

The amount of LTA complexed to surface proteins was determined by trypsinizing the bacteria and calculating the concentration of LTA in the extract using the competitive inhibition ELISA assay. The amount of LTA obtained by trypsinizing the parental strains varied and ranged from a low of 4.8 µg for the M1 serotype to 11.3 µg for the M4 serotype (Table 1). Similarly, the amount of LTA released into the culture media also varied (5.0 to 9.5 µg/ml), as did the amount of membrane-bound LTA in the parental strains (23.8 to 57 µg) (Table 1). Interestingly, the average amount of membrane-bound LTA was significantly less in pattern A serotypes than in non-pattern A serotypes (33.1±9.7 vs 54.7±2.2 µg, p = 0.002).

**Figure 6:**
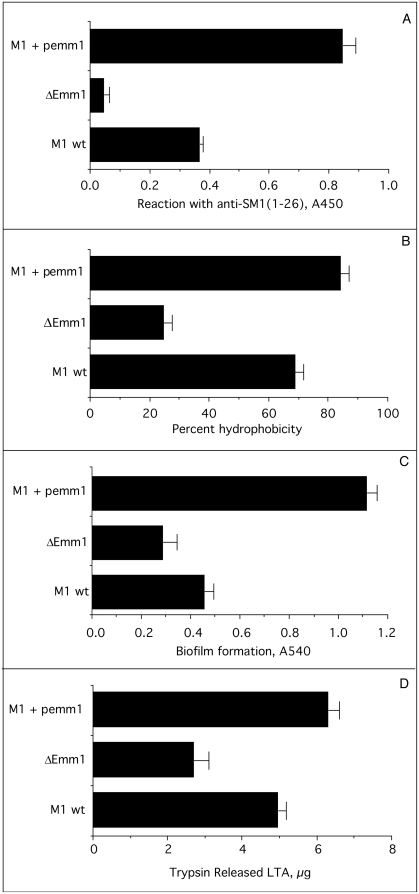
Correlation of membrane-bound LTA with trypsin extracted LTA and with LTA released into the culture media. The amount of LTA bound to membranes was compared to the amount of LTA released into the culture media (A) and to the amount of LTA extracted with trypsin (B). There was a significant degree of correlation in each case, r = 0.730.

Because the M protein family represents some of the most abundant proteins on the surface, we tested mutants defective in expression of these proteins in various serotypes for their role in forming complexes with LTA. In pattern A serotypes, ablation of *emm1*, *emm5*, *emm6*, and *emm24* resulted in a decrease in the amount of protein-bound LTA (i.e. trypsin-extractable) by 65%, 67%, 73%, and 46% respectively (Table 1, Fig. 4). There were no significant differences in the amount of membrane-bound LTA between mutants and parental strains indicating that these mutations only affected the protein-bound LTA and did not alter the amount of membrane-bound LTA. Inactivation of members of the M protein family did not have any consistent effect on the amount of the LTA released into the culture medium as some mutants exhibited a slight increase, or a decrease, or no change (Table 1). However, the amount of LTA released into the culture media did increase as the amount of membrane-bound LTA increased (Fig. 6A). Thus, it appears that the amount of LTA released into the culture medium is primarily dependent upon the concentration of membrane-bound LTA and not upon the manner in which it is complexed to the surface proteins of the streptococci. This is most likely due to the fact that the amount of LTA bound to the surface via protein interactions is quite small compared to the amount of membrane-bound LTA. We suggest that, as LTA is released from the membrane, some of it is bound to surface proteins, but a large portion of the released LTA transits the surface into the culture medium. Thus, membrane-bound LTA serves as a reservoir for protein-bound LTA on the surface and for LTA released into the external milieu.

The membrane-bound LTA does not contribute to hydrophobicity as its lipids are buried within the membrane bilayer of the bacterium. Thus, the membrane-bound LTA has no direct impact on hydrophobicity or biofilms other than serving as a reservoir for LTA that is released into the media or bound to surface proteins. This concept is supported by the finding that the amount of LTA bound to surface proteins and the amount of LTA released into the culture media is directly related to the amount of membrane-bound LTA (Fig. 6). The important point is that it is precisely the protein-bound LTA on the surface that directly influences hydrophobicity and biofilm formation and not the membrane-bound LTA.

In M type 4 *S. pyogenes*, a pattern E serotype, inactivation of *emm*, *mrp*, or *enn* resulted in a 0%, 52%, and 33% reduction in trypsin-extractable LTA respectively (Fig. 5), suggesting the Mrp and Enn but not Emm play a major role in forming complexes with LTA in this serotype. However, inactivation of *emm* and *mrp* in M types 2 and 49 or *emm* and *spa* in M type 18 had no significant effect on the amount of trypsin-extractable LTA (Fig. 5), indicating that other trypsin-sensitive proteins may serve to anchor LTA on the surface in these serotypes.

### Effect of variable expression of M protein on hydrophobicity and biofilm formation

**Figure 7:**
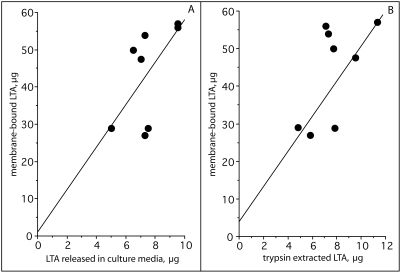
Effect of variable expression of Emm1 on hydrophobicity, biofilm formation, and protein-bound LTA. A. Microtiter wells were coated with the indicated streptococci and then reacted with rabbit anti-SM1(1-26) serum followed by peroxidase-conjugated, goat anti-rabbit Ig. B. The hydrophobicity of the streptococci was determined by adhesion to hexadecane as described in Materials and Methods. C. The ability of the streptococci to form biofilms was determined by the microtiter assays as described in Materials and Methods. D. The amount of protein-bound LTA (trypsin extractable) for the streptococci was determined as described in Materials and Methods. All assays were done in triplicate and the S.D. is shown.

The data above indicate that M proteins are involved in both hydrophobicity and biofilm formation. To determine if the levels of expression of M proteins could have an impact on these functions, a recombinant strain that overexpresses *emm1* was engineered by introducing a plasmid bearing the *emm1* gene (p*emm1*) into the wild type M1 strain of *S. pyogenes*. We then compared the levels of surface expression of Emm1 of this construct to its wild type parent and the Emm1 knock out using antisera against the N-terminal 26 amino acids of Emm1 (Fig. 7A). The ELISA values were 0.365±.011 for wild type, 0.045±0.16 for the ΔEmm1, and 0.845±.041 for the wild type parent containing p*emm1*. Inactivation of *emm1* reduced the binding of anti-SM1(1–26) serum by 95%. Overexpression of Emm1 increased the binding of anti-SM1(1–26) serum by 231% indicating a more than two-fold increase in the expression of Emm1. Thus, we have three isogenic strains with three different levels of Emm1 on their surfaces and these strains were tested for hydrophobicity, biofilm formation, and protein bound LTA (Fig. 7B–D). The results indicate that hyper-expression of Emm1 enhanced the amount of protein bound LTA as well as hydrophobicity and biofilm formation. Conversely, decreased expression of Emm1 not only led to a decrease in the amount of LTA released by trypsin but also to a decrease in hydrophobicity and biofilms. These findings suggest that variations in the amount of M proteins expressed on the surface of streptococci can have a direct impact on the amount of protein-bound LTA, hydrophobicity and biofilm formation.

## Discussion

*S. pyogenes* colonize the human skin and the oral cavity and may stimulate mild to severe local inflammatory responses resulting in pharyngitis in the throat and impetigo in the skin. In susceptible hosts, these infections may lead to life-threatening complications such as sepsis, necrotizing fasciitis, and toxic shock, or to debilitating sequelae such as rheumatic fever and glomerulonephritis. Adhesion and subsequent colonization by *S. pyogenes* has been attributed to a number of surface exposed molecules including members of the M protein family [33], [34]. It has been shown that LTA is also involved in adhesion and that M protein can form complexes with LTA that stabilize this amphipathic molecule in an orientation that exposes its lipids to the external milieu [11], [17]. Such an orientation would allow the lipid moiety of LTA to interact with hydrophobic surfaces of host cells and tissues.

M proteins from pattern A serotypes have multiple functions such as conferring resistance to phagocytosis and binding host proteins that include albumin, fibrinogen, immunoglobulins, and complement regulatory proteins. However, these functions appear to be divided among members of the M protein family in patterns B–E. For example, Mrp binds fibrinogen and confers resistance to phagocytosis, while Emm binds the complement regulator, C4 binding protein (C4BP) [35], [36]. Both Emm and Mrp in pattern B–E serotypes bind immunoglobulins. Enn can also bind immunoglobulins and C4BP [37]. Because M protein from one pattern A serotype was found to be involved in hydrophobicity, this investigation was undertaken to determine if other members of the M protein family may also contribute to hydrophobicity and to the formation of biofilms.

To begin our studies, we trypsinized streptococci from a variety of serotypes to determine if the hydrophobicity and biofilm formation were dependent upon surface proteins. All of the parental strains lost the ability to adhere to hexadecane and to form biofilms after trypsinization, indicating that surface proteins were required for these functions. Next, we used defined mutants to investigate the role that various surface proteins may have in anchoring LTA and contributing to the hydrophobic surface properties and forming biofilms. The ablation of M protein expression in M types 1, 5, 6 and 24 that harbor only a single member of the M protein family indicated that M protein is critically important in conferring hydrophobicity and biofilms in pattern A serotypes. Cho and Caparon [38] also reported that inactivation of *emm* in one pattern A serotype resulted in a significant decrease in biofilm formation. Our finding that the amount of LTA retained on the surface of the mutant strains was greatly diminished compared to that retained by the parent strains suggests that M protein is the major protein in pattern A serotypes that stabilizes LTA on the bacterial surface. Thus, the formation of LTA-M protein complexes appears to be centrally involved in hydrophobicity and biofilm formation in pattern A serotypes.

Other streptococcal surface antigens besides M protein have been found to promote biofilm formation in an M type 1 strain of *S. pyogenes*. Manettii et al. [39] found that inactivation of either the pilus backbone or its sortase decreased both adhesion to host cells and biofilm formation. Our data are consistent with this finding as inactivation of *emm1* led to a partial decrease in biofilm formation. Thus, it appears that both M protein and pilus proteins have a role in the formation of biofilms in M type 1 *S. pyogenes*. This is likely to be the case for other serotypes of *S. pyogenes* that also produce pili.

In M type 4 *S. pyogenes*, a pattern E serotype, there was a direct correlation between hydrophobicity, trypsin-extractable LTA, biofilm formation, and expression of *mrp*. In other serotypes (M2, M18, and M49) from patterns C–E, there was no correlation between hydrophobicity, trypsin-extractable LTA and biofilm formation. The finding that Mrp2 appeared not to be involved in biofilm formation, whereas both Mrp4 and Mrp49 were involved in biofilms was somewhat unexpected because the primary structure of Mrp is highly conserved among serotypes with sequence similarities ranging from 81% to 97% [36]. Whether the lack of involvement by Mrp2 is due to a difference in sequences or to the expression of another protein that is functionally redundant with Mrp2 remains to be determined.

Spa is an antiphagocytic protein and protective antigen that is expressed by a limited number of serotypes [18], [19]. The C-terminus of Spa18 is virtually identical to the C-terminus of Emm from *S. equi*, a horse pathogen [18], suggesting that Spa may also be a member of the M protein family. Although Spa had no role in hydrophobicity, it was a major contributor to biofilm formation in an M type 18 serotype. Thus, Spa shares two functions with members of the M protein family; an antiphagocytic function and a function in biofilm formation. SilC has also been shown to be involved in biofilm formation in M type 18 *S. pyogenes*, but this is presumed to be an indirect effect resulting from the role of SilC as a regulator of streptococcal virulence determinants [26], [40].

These data suggest that the formation of complexes between M proteins and LTA directly contributes to both hydrophobicity and to biofilm formation in most serotypes of *S. pyogenes*. However, in some of the serotypes tested, we were not able to demonstrate a direct link to the M protein family. It may be that in these particular serotypes inactivation of only a single member of the M protein may not be sufficient to alter these functions when another family member is expressed. Alternatively, these serotypes may express a protein that is better suited than M proteins to form complexes with LTA in a manner that allows the lipids of LTA to interact with environmental surfaces.

There are other variables that could influence the contributions of the M protein family to hydrophobicity and biofilm formation. Our data with overexpression of Emm1 and with the Emm1 knock out clearly indicate that alterations in the expression levels of M proteins can have a significant impact on these functions. Thus, there may be variations in the degree of expression of Mrp, Emm or Enn in serotypes from patterns C, D, and E that could also alter the hydrophobicity of these strains and their ability to form biofilms. For example, Northern blot analyses indicated that *emm49* is expressed 4-fold higher than *mrp49* and 16 to 32-fold higher than *enn49* [41]. However, as Mga and its binding sites vary for these genes among different serotypes, it is likely that the relative expression levels of these genes will vary as well. Determining the relative expression levels of Emm, Mrp, and Enn, between and among serotypes and its impact on hydrophobicity and biofilm formation is an important but complex issue that will require future investigations.

Our findings raise questions about how M proteins and LTA contribute to biofilm formation. We suggest that in the case of pattern A serotypes, M proteins contribute by several different mechanisms. The first is by forming complexes with LTA that enhance the hydrophobic surface properties of the streptococci, thereby facilitating interactions with animate and inanimate surfaces. The second is by enhancing interactions between the streptococcal chains by virtue of their N-termini that promote aggregation of the bacteria [42]. The third mechanism is by mediating adhesion to cells [34], thereby, facilitating the initiation of biofilm formation. In those serotypes where inactivation of members of the M protein family had no effect on hydrophobicity but did alter biofilm formation, it is proposed that M proteins and LTA contribute separately to the biofilm formation. Thus, LTA would be complexed to a protein(s) other than M proteins and confer hydrophobicity, whereas one or more members of the M protein family would enhance biofilm formation by mediating adhesion to cells and bacterial aggregation.

The dependence of surface exposed LTA on a specific class of protein is intriguing from the evolutionary point of view and our understanding of *S. pyogenes* virulence. It is known that, although there are variations in M proteins, there are functional constraints that limit the degree of variation. Some of these functional constraints may include binding fibrinogen, albumin, immunoglobulins, and a number of complement regulatory proteins [43]. Our data suggest that the ability to complex with LTA may be an additional function that limits variability in these surface proteins.
