# Correction: Relationship between Expression of the Family of M Proteins and Lipoteichoic Acid to Hydrophobicity and Biofilm Formation in *Streptococcus pyogenes*

**DOI:** 10.1371/annotation/823215e6-c06a-4d5f-ac7b-0ee77067ccb2

**Published:** 2009-01-23

**Authors:** Harry S. Courtney, Itzhak Ofek, Thomas Penfound, Victor Nizet, Morgan A. Pence, Bernd Kreikemeyer, Andreas Podbielski, David L. Hasty, James B. Dale

Figures 6 and 7 appear in the wrong order. The image for Figure 6 should Figure 7, and vice-versa. The figure captions appear in the correct order, but the figures themselves have been switched.

