# Correction: Relationship between Expression of the Family of M Proteins and Lipoteichoic Acid to Hydrophobicity and Biofilm Formation in *Streptococcus pyogenes*

**DOI:** 10.1371/annotation/c2f3e4e5-b6a0-4be8-9f8c-3d4e01933385

**Published:** 2009-01-26

**Authors:** Harry S. Courtney, Itzhak Ofek, Thomas Penfound, Victor Nizet, Morgan A. Pence, Bernd Kreikemeyer, Andreas Podbielski, David L. Hasty, James B. Dale

The seventh author's name was spelled incorrectly. It should read: Andreas Podbielski.

